# Ailments of Voice Users1Extracts from a paper read before the Surgical section, Buffalo Academy of Medicine, December 3, 1895.

**Published:** 1897-07

**Authors:** Horace Clark

**Affiliations:** Buffalo


### AILMENTS OF VOICE USERS.[1]

By HORACE CLARK, A. B., M. D., of Buffalo.

THE object of this paper is to consider the value of cultivation of the voice as a prophylactic measure; as an adjunct to treatment of diseases in the untrained voiceuser ; and to point out a few of the commoner hindrances to successful study in professional singers and speakers. The argument is of general importance as well ; for these diseases are not restricted to voiceusers, as that term is ordinarily understood; nor does every case fall to the special surgeon to treat. Specialists in this department of surgery will insist that in such cases results are far better when patients will study and practise the fundamentals of voice cultivation, subsequently to operative procedure. By this I would be understood to mean the proper adaptation of part to purpose : each part must do only and properly its own allotted task, and must render its own work and that of other parts easier and more perfect by being fixed and free to work as a component and essential part of one nicely adjusted piece of machinery. Refinements of voice cultivation are a luxury, except to the professional, and unnecessary for practical purposes.

### RULES FOR BREATHING.

There are three different kinds of breathing:

(1) Diaphragm breathing, (2) rib breathing, (3) collar-bone breathing. Diaphragm and rib breathing constitute the right way; collar-bone breathing is totally wrong.

*Exercise No. 1.*—(1) Lie upon the back. (2) Inhale slowly through the nostrils. The abdomen will rise, the lower part of the chest will expand sideways, and the upper part will be pushed forward. (3) Now hold the breath, not by closing the glottis or voice-box, but by keeping the diaphragm down. (4) Count four, at the rate of about sixty per minute, and let the breath go *suddenly*. Increase the length of time of holding the breath two seconds per week, not exceeding twelve seconds as a maximum limit.

(1) Inhale rapidly through the nostrils. (2) Do not hold the breath, but expire slowly and evenly. A test of successful expiration is to breathe against a candle-flame, which should not wave to and fro, but be blown steadily away.

1. Extracts from a paper read before the Surgical section, Buffalo Academy of Medicine, December 3, 1895.

(1) Inspire slowly. (2) Expire slowly. This is easily done, the first two exercises having been learned.

### VOICE FATIGUE.

Have plenty of breath, and have it under control. Commence to expire only at the moment when the vocal cords are to be set in vibration. Never use the voice when there is indication of its impairment. In singing or speaking, avoid inclement weather, or facing a stormy wind, and foul air and noise. Do not exhaust all the air taken in at one inspiration before beginning another.

### ATTACK.

By this is meant the exact approximation of the vocal cords at the moment of phonation. The voice-box is opened and closed as follows :

*Exercise No. 2.*—(1) In the pitch of the spoken voice sing the combinations OO, AH, AI, EE, and follow each one by a short inspiration.

In this exercise avoid two things, (1) "Glide," (2) "Click" of the Glottis. This is done as follows : (1) Pronounce the word "UP" vigorously. (2) Whisper the vowel "U" as pronounced in "UP." Then sing "AH." In singing these tones as suggested, it is important not to overcrowd the lungs. The remedy for this is to sing a few tones without inspiring.

### RESONANCE.

This is defined when a hollow shell is held close to the ear. Mistakes made in this matter relate to the mouth, lips, tongue and soft palate. Easy alteration in the position of the jaws and lips is essential as a first principle in studying resonance.

*Exercise No. 3.*—(1) Arrange a mirror so that all the parts can be readily seen. (2) Open the mouth as widely as possible in every direction. (3) Look at the tongue, the soft palate and the back of the throat. (4) Close the mouth again and repeat this procedure several times. (5) Open the mouth widely enough to put two fingers between the teeth ; then smile, so as to draw the corners of the mouth sideways until they are each bordered by a perpendicular line. Now suddenly pucker the lips as if to whistle. (6) Smile with lips firmly closed, drawing the corners of the mouth sideways as much as possible ; then pucker the lips as if to whistle, but leave no aperture. These exercises seem absurd, but they render excellent service.

### CONTROL OF THE TONGUE.

*Exercise No. 4.*—(1) Open the mouth wide. Put out the tongue straight and as far as possible. Draw it back quickly and try to make it lie flat and without tremor, touching the teeth all around, especially the back teeth. (2) Put the tip of the tongue against the lower front teeth, and push it out as far as possible. Then draw it back quickly as in the first exercise. (3) Keep the root of the tongue flat; raise the tip and push it perpendicularly and slowly towards the roof of the mouth. Gradually lower it again until it has assumed its original position. (4) Raise the tip of the tongue as in 3, and move it gradually from side to side, so that its highest point will describe a semi-circle.

### GYMNASTICS OF THE SOFT PALATE.

The soft palate can be raised or lowered so that the throat can be completely shut off from the nose or mouth.

*Exercise No. 5.*—(1) Breathe through the mouth; the soft palate will be moderately raised, the uvula retaining its normal shape and position. In expiration through the mouth, the uvula will be thrown a little forward. (2) Open the mouth again, and try to breathe through the nose. The soft palate will fall, and the tongue will rise a little. Exhale in the same way, and the mouth will shut at the back. (3) Inhale through the nostrils, the mouth being wide open. Prevent the tongue from rising. The soft palate will now come down more firmly. Exhale through the mouth, and the soft palate will rise again.

These exercises greatly strengthen the muscles of the soft palate and the benefit to the voice is incalculable.

"Flexibility" relates to tightening or relaxing the vocal cords. A "Register" consists of a series of tones produced by the same mechanism, and there are five "registers," but these are matters which cannot be included in a tabular view of voice exercises.

The above was supplemented by illustration and explanation.

In conclusion, I would call the lay-reader's attention to the fact that the site of disease is usually fixed by the patient where the trouble is felt, whereas in a large majority of cases of loss of the singing or speaking voice, for example, the larynx proper escapes injury unless, indeed, there is coëxistant constitutional mischief. Such cases are usually caused by digestive disturbances, or faulty mechanism in the resonating cavities which has been strained, or whose membranes has become acutely inflamed.

A source of trouble to the voiceuser, not infrequently overlooked, is to be found in the glands at the base of the tongue. I have seen many cases in which extensive intranasal surgery was done to relieve obstinate cough, without result; whereas the removal of hypertrophied glands at the base of the tongue gave immediate and permanent cure.

846 ELLICOTT SQUARE.

